# Risk of progression following a negative biopsy in prostate cancer active surveillance

**DOI:** 10.1038/s41391-022-00582-x

**Published:** 2022-08-25

**Authors:** Kerri Beckmann, Aida Santaolalla, Mikio Sugimoto, Peter Carroll, Jose Rubio, Arnauld Villers, Anders Bjartell, Todd Morgan, Prokar Dasgupta, Mieke Van Hemelrijck, Oussama Elhage, The Movember Foundation’s Global Action Plan Prostate Cancer Active Surveillance (GAP3) Consortium

**Affiliations:** 1 Cancer Epidemiology and Population Health Research Group, University of South Australia, Adelaide, Australia; 2 Translational Oncology and Urology Research, Kings College London, London, UK; 3 Faculty of Medicine, Kagawa University, Kagawa, Japan; 4 Department of Urology, UCSF—Helen Diller Comprehensive Cancer Centre, University of California San Francisco, San Francisco, CA USA; 5 Instituto Valenciano de Oncologia, Valencia, Spain; 6 Lille University Hospital Center, Lille, France; 7 Department of Urology, Skane University Hospital, Malmo, Sweden; 8 University of Michigan and Michigan Urological Surgery Improvement Collaborative, Michigan, MI USA; 9 Guy’s and St Thomas’ NHS Foundation Trust, London, UK; 10 Immunology and Microbial Sciences, King’s College London, London, UK

**Keywords:** Cancer therapy, Outcomes research, Prostate cancer

## Abstract

**Background:**

Currently, follow-up protocols are applied equally to men on active surveillance (AS) for prostate cancer (PCa) regardless of findings at their initial follow-up biopsy. To determine whether less intensive follow-up is suitable following negative biopsy findings, we assessed the risk of converting to active treatment, any subsequent upgrading, volume progression (>33% positive cores), and serious upgrading (grade group >2) for negative compared with positive findings on initial follow-up biopsy.

**Methods:**

13,161 men from 24 centres participating in the Global Action Plan Active Surveillance Prostate Cancer [GAP3] consortium database, with baseline grade group ≤2, PSA ≤ 20 ng/mL, cT-stage 1–2, diagnosed after 1995, and ≥1 follow-up biopsy, were included in this study. Risk of converting to treatment was assessed using multivariable mixed-effects survival regression. Odds of volume progression, any upgrading and serious upgrading were assessed using mix-effects binary logistic regression for men with ≥2 surveillance biopsies.

**Results:**

27% of the cohort (*n* = 3590) had no evidence of PCa at their initial biopsy. Over 50% of subsequent biopsies in this group were also negative. A negative initial biopsy was associated with lower risk of conversion (adjusted hazard ratio: 0.45; 95% confidence interval [CI]: 0.42–0.49), subsequent upgrading (adjusted odds ratio [OR]: 0.52; 95%CI: 0.45–0.62) and serious upgrading (OR: 0.74; 95%CI: 0.59–92). Radiological progression was not assessed due to limited imaging data.

**Conclusion:**

Despite heterogeneity in follow-up schedules, findings from this global study indicated reduced risk of converting to treatment, volume progression, any upgrading and serious upgrading among men whose initial biopsy findings were negative compared with positive. Given the low risk of progression and high likelihood of further negative biopsy findings, consideration should be given to decreasing follow-up intensity for this group to reduce unnecessary invasive biopsies.

## Introduction

Active Surveillance (AS) is the current standard management strategy for men with low risk or favourable intermediate risk prostate cancer (PCa) to reduce overtreatment of what is likely to be indolent disease. While surveillance protocols vary across countries and between centres, most involve ongoing PSA monitoring and physical examination at 6–12 monthly intervals and repeat biopsy initially at or before 12 months and then regularly at 1–4 yearly intervals [1, 2]. While the aim of AS is to reduce morbidity and side effects associated with radical treatments, invasive monitoring via repeat biopsy can be emotionally and physically distressing, and is not without risk, with infection rates following biopsy around 5% depending on biopsy technique [3]. Magnetic resonance imaging (MRI) is being used increasingly in AS follow-up protocols to identify new lesions and monitor changes in existing lesions. While MRI is a useful addition, at present, evidence still suggests it cannot replace biopsy and thus histological confirmation is still recommended [4].

Several studies indicate that risk of disease progression while on AS is considerably lower for men who have negative compared to positive findings at their initial biopsy despite cancer being detected at the diagnostic biopsy. Studies in single institutions [5–8] and in a multicentre cohort [9], have reported between 50–62% reduced risk of disease progression in men whose initial biopsy was negative compared to those with a positive biopsy. These findings suggest a less intense surveillance protocol may be suitable for men with negative biopsy findings.

This study investigated the risk of transitioning to treatment and risk of any upgrading and serious upgrading (grade group >2) following negative compared with positive findings at the initial AS biopsy, within the Movember Foundations’ Global Action Plan Active Surveillance Prostate Cancer database (GAP3), the largest multi-national database of men on AS [10]. Our secondary aim was to identify predictors of upgrading among men who had negative biopsy findings, to inform recommendations around tailoring surveillance schedules to be less intense for this group.

In this study we examined risk of upgrading and transitioning to treatment depending on the outcome of the initial biopsy (negative vs positive for PCa) using data from the Movember Foundations’ Global Action Plan Active Surveillance Prostate Cancer database (GAP3), the largest multi-national database of men on AS [10]. Addressing these questions in GAP3, which documents the experiences of such a large number of men from multiple centres undergoing AS for PCa, is important in providing confirmatory evidence on a global scale to guide practice among those who have negative biopsy findings during follow-up.

## Patients and methods

### Study objectives

Our overall objective was to provide evidence to inform policies and practice around tailoring surveillance schedules for men on AS for PCa who have negative biopsy findings at their initial surveillance biopsy. This study’s primary aim was to assess the risk of transitioning to treatment, risk of any upgrading and serious upgrading (grade group >2) following a negative compared with positive findings at the initial biopsy among men with PCa. Our secondary aim was to identify predictors of upgrading among men who had negative biopsy findings.

### Study cohort

Study participants were drawn from the GAP3 database (version 3.2, November 2019). Centres with an AS database registering 50 or more patients annually were invited to contribute deidentified unit record data to a central platform according to standardised reporting template, with individual institutional ethical approval [10]. The GAP3 v3.2 included approximately 21,000 men from 27 centres across Europe, North America, Asia and Australia.

The following inclusion criteria applied: diagnosis on or after Jan 1, 1995, low to intermediate risk disease (Grade Group <3 at diagnostic biopsy, T-stage<cT3, PSA < 20 ng/ml) and at least one post-diagnostic biopsy. Men managed at two centres with follow-up protocols that included MRI triggered biopsy only were also excluded. The eligible cohort consisted of 13,161 men from 24 centres. Participant selection is shown in Supplementary Fig 1.

### Measures

Men were classified according to findings at their first biopsy (irrespective of its timing) as having a negative or positive prostate biopsy. Findings were classified as negative if records indicated no biopsy cores containing cancer and no other indication of the presence of cancer. Findings were classified as positive if there was any evidence of cancer based on reported number of positive cores, maximum cancer core length, primary or secondary Gleason score, or the percentage of cancer present at their initial follow-up biopsy.

Outcomes assessed included: (a) risk of converting to treatment (including curative and non-curative therapies); (b) risk of any upgrading from biopsy grade (i.e., grade group 1 to grade group ≥2, or grade group 2 to grade group >2) at a subsequent biopsy; (c) risk of upgrading to grade group >2 (serious upgrading) at a subsequent biopsy; (d) risk of PCa volume progression (i.e., percent of positive cores>33%; and (e) risk of volume or grade progression.

### Statistical analysis

Risk of conversion following their initial biopsy was assessed in all eligible men using mixed-effects survival regression analyses, with Weibull distribution and random intercept for treatment centre to account for potential heterogeneity between centres. Follow-up time commenced from date of their initial biopsy until conversion to active treatment, with censoring at the date of death, conversion to watchful waiting (i.e., no active follow-up and non-curative management if symptoms developed) or last known follow-up appointment. Survival models were adjusted for characteristics at baseline including age at diagnosis (5-year age groups), year of diagnosis (5-year periods), biopsy grade group, Clinical T-stage, baseline PSA ( < 5, 5–9.9, 10–14.9, 15–20 ng/ml), prostate volume (continuous per 5 cc), number of cores sampled at biopsy (continuous), number of cores positive at biopsy (continuous), and time interval to initial biopsy (continuous per year). Missing data for covariates were imputed using multiple imputation chained equations. Data on biopsy approach and MRI use during diagnostic work-up were unavailable.

Risk of any upgrading, serious upgrading and volume progression at any subsequent biopsy were assessed via mixed-effects binary logistic regression. These models were adjusted for the same covariates as above and only included men who underwent at least one further follow-up biopsy (*n* = 6638). All above mentioned models included a single record per individual with random intercepts for treatment centre.

To identify potential predictive factors for any subsequent upgrading or for serious upgrading after a negative biopsy, mixed-effects logistic regression was undertaken within the subset of men who had negative findings who had undergone further follow-up biopsies (*n* = 2164). Model fit was assessed using Akaike Information Criteria, with respect to inclusion of the above-mentioned covariates, as well as clinical characteristics at their initial biopsy (number of cores sampled, PSA, PSA density).

### Sensitivity analyses

Further sensitivity analyses were undertaken which assessed: risk of conversion to treatment, only among men who underwent at least one further follow-up biopsy (*n* = 6638); and odds of upgrading/serious upgrading/volume progression at any subsequent biopsy, modelling all biopsy records after the initial biopsy (*n* = 13,468, i.e., multiple records per individual). The last set of models included random intercepts for individual participants and for treatment centre.

## Results

### Cohort description

**Fig. 1:**
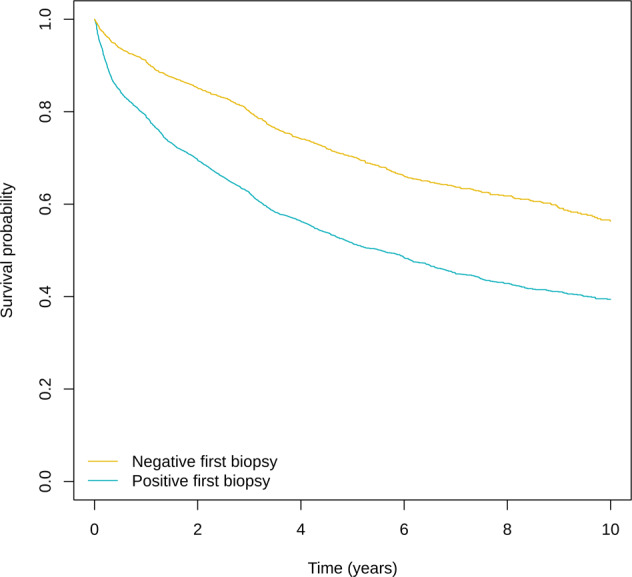
Risk of transitioning to treatment while on active surveillance according to whether initial follow-up biopsy findings were positive or negative for prostate cancer.

Of the 13,161 eligible men, 3590 (27%) had negative findings at their initial biopsy (i.e., no evidence of PCa present). Clinical characteristics at diagnosis were similar between positive and negative biopsy groups for median age, PSA level, number of cores sampled, and number of positive cores (Table 1). The proportions with cT2 and Grade Group 2 disease were slightly lower and median prostate volume slightly higher among the negative biopsy group. Time on AS for the cohort overall was 3.2 years (range 0.34–14.4 years), but this differed between groups, with the negative biopsy group having longer time in AS, and hence, a greater number of subsequent biopsies (59% had ≥2 follow-up biopsies vs 37% among the positive group). Median time to initial biopsy was 1.0 years for both the positive and negative group, while median time between first and second biopsy was 1.3 and 1.8 years, respectively. The proportions still on AS after 5 years of follow-up were 26.2% and 41.2% for the positive and negative biopsy group, respectively, while 38.7% vs 22.2% had converted to treatment (Table 1). Kaplan-Meier plots for conversion to treatment are shown in Fig. 1.

**Table 1:** Characteristics of study cohort, by confirmatory biopsy status.

	Positive Biopsy (*n* = 9571)		Negative Biopsy (*n* = 3590)		*p*-value
Diagnostic characteristics^**a**^					
Median age (IQR)	65	(60–70)	65	(60–69)	0.33
Median PSA (IQR)	5.4	(4.2–7.1)	5.4	(4.1–7.1)	0.49
Gleason grade (*n*, %)					
grade group 1	9016	94.2	3540	98.6	<0.001
grade group 2	555	5.8	50	1.4	
Clinical T stage (*n*, %)					
cT1	7995	83.5	3150	87.7	<0.001
cT2	1576	16.5	440	12.3	
Median no. cores sampled (IQR)	12	(10–12)	12	(10–13)	0.01
Median no cores positive (IQR)	1	(1–2)	1	(1–2)	<0.001
Median prostate volume (IQR)	44	(34–57)	47	(37–60)	<0.001
Follow-up characteristics					
Median follow-up time, yrs (IQR)^b^	2.7	(1.3–5.1)	4.6	(2.8–7.1)	<0.001
Median time to first biopsy, yrs (IQR)	1.0	(0.3–1.2)	1.0	(0.7–1.1)	<0.001
Median interval biopsy 1 to 2, yrs (IQR)	1.3	(0.8–2.2)	1.8	(1.0–3.0)	<0.001
Median PSA prior to first biopsy (IQR)	5.6	(4.0–7.7)	5.2	(3.4–7.2)	<0.001
Median no. cores taken at first biopsy (IQR)	12	(10–14)	12	(10–14)	0.02
Median no. follow-up biopsies (IQR)	1	(1–2)	2	(1–3)	<0.001
Underwent ≥2 follow-up biopsies (*n*, %)	4475	37.0	2163	59.5	<0.001
AS status at 5 yrs (n, % total):					
Converted to active treatment	3613	38.7	796	22.2	<0.001
Still on AS	2503	26.2	1478	41.2	
Censored <5 yrs while still on AS	3314	34.6	1136	31.6	
Switch to watchful waiting	101	1.1	47	1.3	
Lost to follow-up	117	1.2	97	2.7	
Died	66	0.7	36	1.0	
Reported reasons for conversion (*n*, %)^c^	*N* = 3966	41.4	*N* = 1038	28.5	
Pathological progression	2089	21.8	493	13.7	<0.001
Clinical or radiological progression	266	2.8	57	1.6	
PSA progression/kinetics only	172	1.8	65	1.8	
Patient choice	283	2.9	125	3.5	
cT Unknown/not reported	1156	12.1	268	8.3	
Upgrading at 2nd follow-up biopsy (*n*, %)	*N* = 4475		*N* = 2163		
Any upgrading	936	27.8	211	14.0	<0.001
Serious upgrading	309	6.9	91	4.2	

% missing data: age > 0.6%; PSA = 2.7%; +ve cores = 4.2%; cT = 10.9; Prostate Volume = 20.9%.

^a^data were imputed using the MICE module in Stata.

^b^time from diagnosis to end of AS surveillance/follow-up.

^c^among all men who converted to treatment at any time during AS.

**Fig. 2:**
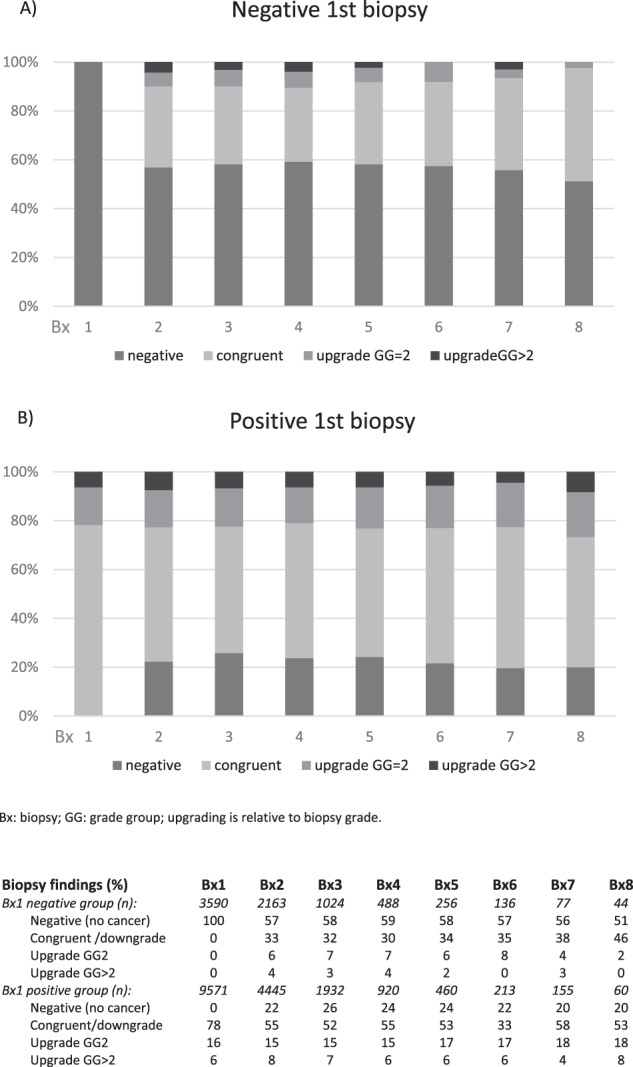
Grading classification at each repeat biopsy according to initial follow-up biopsy findings among men on active surveillance. **A** Negative 1st biopsy. **B** Postivive 1st biopsy. Bx biopsy, GG Grade group.

Outcomes of repeat biopsies are presented for those with positive and negative findings at initial biopsy in Fig. 2. Among the negative biopsy group, >50% of findings at each subsequent biopsy were also negative for PCa (up to 8 repeat biopsies). Overall, 41% of men in the negative biopsy group with ≥3 biopsies had persistent negative findings. The proportion of subsequent biopsies where any upgrading occurred, including serious upgrading, remained low.

### Multivariable analyses of outcomes

After adjusting for baseline clinical characteristics, risk of converting to treatment was significantly lower among men who had negative compared with positive findings at their initial biopsy (hazard ratio [HR]: 0.45; 95% confidence interval [CI]: 0.42–0.49). (Table 2, full model output provided in Supplementary Table 1) In sensitivity analysis, which included only men who had ≥2 biopsies, the HR for conversion to treatment was 0.65 (95% CI: 0.58–0.72) for negative compared with positive initial biopsy.

**Table 2:** Risk for transitioning to treatment, upgrading and volume progression for negative versus positive findings at first follow-up (confirmatory) biopsy among men on active surveillance for prostate cancer.

Outcomes/model [sensitivity analyses based on different modelling approaches]	Hazard/Odds ratio for negative vs positive biopsy		
Conversion to treatment	HR	95% CI	*p*-value
men who underwent > =1 biopsy (*n* = 13,161 men)	0.45	0.42–0.49	<0.001
[men who underwent > =2 biopsies (*n* = 6638 men)]	0.65	0.58–0.72	<0.001
Any upgrading	OR	95% CI	*p*-value
at any subsequent biopsy (*n* = 6638 men)	0.52	0.45–0.60	<0.001
[^#^at any subsequent biopsy, per biopsy event (*n* = 13,468 biopsies)]	0.31	0.24–0.39	<0.001
Serious upgrading (grade group>2)	OR	95% CI	*p*-value
at any subsequent biopsy (*n* = 6638 men)	0.74	0.59–0.92	0.007
[^#^at any subsequent biopsy, per biopsy event (*n* = 13,468 biopsies)]	0.61	0.46–0.80	<0.001
Volume progression (>33% positive cores)	OR	95% CI	*p*-value
at any subsequent biopsy (*n* = 6638 men)	0.34	0.28–0.42	<0.001
[^#^at any subsequent biopsy, per biopsy event (*n* = 13,468 biopsies)]	0.20	0.15–0.26	<0.001
Volume and (any) grade progression	OR	95% CI	*p*-value
at any subsequent biopsy (*n* = 6638 men)	0.42	0.37–0.49	<0.001
[^#^at any subsequent biopsy, per biopsy event (*n* = 13,468 biopsies)]	0.25	0.20–0.31	<0.001

*CI* confidence intervals, *HR* hazard ratio derived from multivariable mixed effects survival regression models, *OR* Odds ratios derived from multivariable mixed effect logistic regression models - random intercept for treatment centre.

^#^models include multiple biopsy records and random intercept for treatment centre and for each study participant

Odds of subsequent upgrading (odds ratio [OR]: 0.52; 95% CI: 0.45−0.60) and subsequent serious upgrading (OR: 0.74; 95% CI: 0.59−0.92) were also reduced among the negative biopsy group. Odds of subsequent volume progression (OR: 0.34; 95% CI: 0.28−0.42) and progression based on volume or any upgrading (OR: 0.42; 95% CI: 0.37−0.49) were also reduced among those whose initial biopsy was negative compared with positive for PCa. Models which assessed upgrading/volume progression per biopsy event rather than per individual level showed stronger effects. (Table 2, full model output provided in Supplementary Tables 2–3).

### Predictors of upgrading following negative biopsy

Among those who had undergone multiple surveillance biopsies and had negative findings at their initial biopsy, 149 (7%) experienced serious upgrading. Median time from diagnosis to upgrading was 4.0 years (IQR 1.6–6.0 years). In unadjusted analyses, no clinical characteristic clearly distinguished between those who experienced subsequent upgrading/serious upgrading following a negative biopsy and those who did not, with the vast majority having features at diagnosis that would be consider low (or very low) risk for disease progression (Supplementary Table 4).

In multivariable models, the factors associated with serious upgrading following a negative biopsy included: age at diagnosis (OR 2.45; 95% CI: 1.26–4.80 for ≥75 vs <55 yrs), biopsy grade group (OR 3.05; 95% CI: 1.00–9.39 for grade group 2 vs 1) and PSA density at diagnosis (OR 1.03; 95% CI: 1.00–1.06, per 0.01 increment) (Table 3). The overall ability of these baseline characteristics to identify those at risk of serious upgrading following a negative initial biopsy was relatively low (with area under the receiver operating curve = 0.61). PSA density at diagnosis produced a better fitting model than either PSA concentration or prostate volume individually, or simultaneously. The inclusion of follow-up characteristics, with or without diagnostic characteristics, did not improve model fit. In analyses to identify potential predictors of any subsequent upgrading, older age at diagnosis was associated with increased risk (OR 2.45; 95% CI: 1.26–4.80 for ≥75 vs <55 yrs). No other clinical characteristics known at diagnosis or at the initial follow-up biopsy were independently associated with risk of subsequent upgrading.

**Table 3:** Predictors of upgrading (at any subsequent follow-up biopsy) among men with negative confirmatory biopsy.

Factors	Any upgrading			Upgrade to grade group > 2		
	0 R	95% CI	*p*-value	0 R	95% CI	*p*-value
Age [Ref: <55 yrs]	1.00	ref	–	1.00	ref	
55–59	1.32	0.80–2.18	0.277	1.21	0.54–2.70	0.639
60–64	1.37	0.86–2.19	0.178	1.46	0.70–3.02	0.312
65–69	1.66	1.05–2.62	0.027	1.57	0.77–3.22	0.214
70–74	1.63	1.00–2.67	0.052	2.22	1.05–4.69	0.036
≥75	2.47	1.28–4.76	0.007	2.62	1.00–6.89	0.050
Diagnosis period [Ref: <2004]	1.00	ref	–	1.00	ref	
2005–2009	0.74	0.46–1.19	0.215	0.87	0.42–1.77	0.693
2010–2014	0.60	0.38–0.96	0.033	0.98	0.49–1.96	0.946
2015–2018	0.56	0.32–0.98	0.044	0.72	0.31–1.66	0.441
Grade dx [3 + 4 vs 3 + 3]	1.09	0.38–3.17	0.871	3.08	1.00–9.54	0.050
Stage dx [cT2 vs cT1]	0.86	0.61–1.22	0.408	1.25	0.49–2.00	0.366
No. cores taken at diagnosis [continuous]	0.99	0.97–1.00	0.460	0.98	0.94–1.02	0.380
No. cores positive at diagnosis [continuous]	1.09	0.94–1.25	0.264	1.07	0.88–1.30	0.485
PSA density at diagnosis [continuous]	1.02	1.00–1.04	0.063	1.03	1.01–1.06	0.010
c-statistic			C = 0.63			C = 0.61

*OR* Odds ratios derived from mixed effects logistic regression models restricted to men who had negative findings at first follow-up biopsy and subsequently underwent one or more further follow-up biopsies, with random intercept for treatment centre (*n* = 2159, 1 record per individual).

## Discussion

Our analyses of data from the GAP3 database indicate lower risk of transitioning from AS to active treatment and lower risk of upgrading, including upgrading to grade group >2 and volume progression, following negative compared with positive findings at their initial biopsy. Older age, biopsy grade and higher PSA density at diagnosis were associated with risk of upgrading to grade group >2 among men whose biopsy was negative. However, overall ability to predict serious upgrading based on these characteristics alone was poor.

Our findings are consistent with results from single institutions [5–7, 11] or multi-institutional, single-country studies [9] which have reported 50–70% reduction in risk of disease progression (assessed through upgrading, volume increase or both). In the GAP3 cohort the absolute risk of serious upgrading, indicating the need for radical treatment, was very low among men whose initial biopsy was negative for PCa (3.6% of all subsequent biopsies). In contrast, chances of further negative findings at all subsequent biopsies were high (41%). A recent single-institute study of men enrolled in the University of California San Francisco AS cohort showed extremely low risk of risk of having detectable cancer at the fourth biopsy among those who had consistently negative prior biopsies [12].

While these findings are not novel, their confirmation in the largest international AS dataset signals the need to consider different surveillance protocols for men with negative biopsy findings.

From our data, we estimate that the additional number of biopsies required to detect one case of upgrading among those with initially negative biopsy findings would be 8, while the number required to identify one case of serious upgrading would be 44. Thus, we recommend that consideration of further biopsies only be based on other indications (i.e., increases in PSA density, or concerning changes on imaging if MRI is used in AS follow-up). Avoiding unnecessary biopsies would reduce the burden on both men and health systems when risk of progression is low, with considerable time and cost savings in health care settings and reduced discomfort and distress for patients undergoing the procedure. The small risk of complications following transperineal biopsy would also be avoided completely in this group[13]. Furthermore, compliance with scheduled repeat biopsies has been shown to decline over time [14, 15]. The requirement for repeated biopsy may dissuade some men from continuing on AS despite the lack of evidence of disease progression [16–18].

At a minimum, PRIAS protocols, which specify repeat biopsies at years 1, 4, and 7 [19], could be applied safely to this very low risk population. Risk of upgrading does not appear to vary appreciably for men managed via PRIAS protocols compared with those with more frequent biopsy schedules [20]. Consideration could also be given to adopting risk-based follow-up schedules. While our findings suggested limited ability to accurately predict serious upgrading among those with negative biopsy findings, several risk assessment tools with greater discriminatory ability are available for men on AS [21]. For example, the ‘Canary Active Surveillance Study Risk Calculator’ [22], which predicts grade reclassification based on age at diagnosis, latest PSA concentration, percentage of positive cores and prior number of negative biopsies, has moderately good discriminatory ability (area under receiver operating curve 0.74 in the original test cohort [22] and 0.65–0.68 in external validation datasets [23, 24]). Likewise, modelling risk based on PSA velocity (PRIAS tool) provides moderate discrimination for upgrading in AS cohorts [25], suggesting that monitoring rates of change in PSA may be sufficient for men with negative biopsy findings.

The use of MRI in diagnostic work up and in AS surveillance may provide additional information to improve identification of those at risk of disease progression who require active treatment. Unfortunately, studying the influence of MRI-based surveillance was not possible with available GAP3 data. Whether MRI-based protocols with targeted biopsies, where indicated by changes on imaging, can completely replace the need for standard repeat biopsies in AS is still an area of considerable debate [26, 27] and intense research interest [28, 29]. Some institutional protocols and guideline authorities (e.g., UK’s National Institute of Health and Care Excellence [30]) are already recommending MRI-based surveillance schedules, with biopsy only on indication of new or changed lesions seen on imaging, rather than fixed repeat biopsy schedules. Applying MRI-based follow-up schedules may be an appropriate option for surveillance of men when their initial follow-up biopsy was negative for PCa, though this needs further investigation. The relatively poor predictive value of our modelling also underscores the need for greater emphasis on research to identify useful biomarkers for progression during AS [31].

### Limitations

While GAP3 is a large international, multi-institutional dataset, median follow-up time is relatively short limiting our ability to assess long-term outcomes. Only around 50% of eligible men had undergone multiple surveillance biopsies, limiting the number who could be assessed with respect to upgrading and volume progression. Data on biopsy approach (transrectal or transperineal), which is potentially an important confounder, were not consistently available. In addition, limited data on MRI at diagnostic workup and during surveillance (only reported by 5-centres) prevented us from adjusting for its influence on outcomes. While suspicion on MRI will usually be confirmed by subsequent biopsy (and hence would be captured in our data), the lack of imaging data may limit generalisability of our findings to many modern AS practices in which MRI surveillance plays a major role. However, we believe our findings are still applicable to current practice in many settings where MRI is not available (e.g., in countries that are less well resourced). Similarly, there are situations in which some men cannot undergo MRI. Our findings are also very valuable in the category of patients who originally had PiRAD 3 or less with no specific changes, or no obvious lesion on the MRI and score PRECISE 3 on follow up MRI, as these patients would usually undergo systematic biopsies.

While there was heterogeneity in policies and practices of individual centres regarding repeat biopsy schedules and triggers for transitioning to treatment, the likelihood of systematic biases (and hence false claims of reduced risk) would be very low. On the contrary, we would have expected the effect of heterogenous follow-up schedules to be toward null findings, which serves to strengthen our findings.

## Conclusion

This is the first study to examine outcomes following negative biopsy findings using data from an international database comprising men from multiple centres with varied protocols for AS. Despite this heterogeneity results consistently showed lower risk of upgrading and conversion to treatment following negative compared with positive findings for cancer at the confirmatory biopsy. Likelihood of further negative findings at subsequent biopsies was also high. Consideration should be given to altering follow-up schedules and incorporating less invasive surveillance approaches for men whose confirmatory biopsy findings were negative to reduce or avoid unnecessary invasive biopsies.

## Supplementary information

Appendix A

Supplementary Material

## Data Availability

The datasets generated during and/or analysed during the current study are available from the principal investigator of the GAP 3 consortium, c.h.bangma@erasmusmc.nl, on reasonable request.
