# Supplementary material for: Risk of progression following a negative biopsy in prostate cancer active surveillance
Source: Prostate Cancer Prostatic Dis. 2022 Aug 25;26(2):403–9. doi: 10.1038/s41391-022-00582-x (PMC10247354; doi:10.1038/s41391-022-00582-x)
Supplement: Supplementary file 1 — Appendix A [file 41391_2022_582_MOESM1_ESM.docx]

**Appendix A. Members of The Movember Foundation’s Global Action Plan Prostate Cancer Active Surveillance (GAP3) consortium**

Principle Investigators: Bruce Trock (Johns Hopkins University, The James Buchanan Brady Urological Institute, Baltimore, USA), Behfar Ehdaie (Memorial Sloan Kettering Cancer Center, New York, USA), Peter Carroll (University of California San Francisco, San Francisco, USA), Christopher Filson (Emory University School of Medicine, Winship Cancer Institute, Atlanta, USA), Christopher Logothetis (MD Anderson Cancer Centre, Houston, USA), Todd Morgan (University of Michigan and Michigan Urological Surgery Improvement Collaborative (MUSIC), Michigan, USA), Laurence Klotz (University of Toronto, Sunnybrook Health Sciences Centre, Toronto, Ontario, Canada), Tom Pickles (University of British Columbia, BC Cancer Agency, Vancouver, Canada), Eric Hyndman (University of Calgary, Southern Alberta Institute of Urology, Calgary, Canada), Caroline Moore (University College London & University College London Hospital Trust, London, UK), Vincent Gnanapragasam (University of Cambridge & Cambridge University Hospitals NHS Foundation Trust, Cambridge, UK), Mieke Van Hemelrijck (King's College London, London, UK & Guy’s and St Thomas’ NHS Foundation Trust, London, UK), Prokar Dasgupta (Guy’s and St Thomas’ NHS Foundation Trust, London, UK), Chris Bangma (Erasmus Medical Center, Rotterdam, The Netherlands/ representative of Prostate cancer Research International Active Surveillance (PRIAS) consortium), Monique Roobol (Erasmus Medical Center, Rotterdam, The Netherlands/ representative of Prostate cancer Research International Active Surveillance (PRIAS) consortium), The PRIAS study group, Arnauld Villers (Lille University Hospital Center, Lille, France), Grégoire Robert (Centre Hospitalier Universitaire de Bordeaux (CHU), Bordeaux, France), Axel Semjonow (University Hospital Muenster, Muenster, Germany), Antti Rannikko (Helsinki University and Helsinki University Hospital, Helsinki, Finland), Riccardo Valdagni (Department of Oncology and Hemato-oncology, Università degli Studi di Milano, Radiation Oncology 1 and Prostate Cancer Program, Fondazione IRCCS Istituto Nazionale dei Tumori, Milan, Italy), Antoinette Perry (University College Dublin, Dublin, Ireland), Jonas Hugosson (Sahlgrenska University Hospital, Göteborg, Sweden), Jose Rubio-Briones (Instituto Valenciano de Oncología, Valencia, Spain), Anders Bjartell (Skåne University Hospital, Malmö, Sweden), Lukas Hefermehl (Kantonsspital Baden, Baden, Switzerland), Lee Lui Shiong (Singapore General Hospital, Singapore, Singapore), Mark Frydenberg (Monash University ; Cabrini Institute, Cabrini Health, Melbourne, Australia), Phillip Stricker (St Vincents Prostate Cancer Centre, Sydney, Australia), Mikio Sugimoto (Kagawa University Faculty of Medicine, Kagawa, Japan), Byung Ha Chung (Gangnam Severance Hospital, Yonsei University Health System, Seoul, Republic of Korea)

Pathologist: Theo van der Kwast (Princess Margaret Cancer Centre, Toronto, Canada).

Technology Research Partners: Wim van der Linden, Tim Hulsen, Boris Ruwe, Peter van Hooft (Royal Philips, Eindhoven, the Netherlands).

Executive Regional statisticians: Ewout Steyerberg (Erasmus Medical Center, Rotterdam, The Netherlands), Daan Nieboer (Erasmus Medical Center, Rotterdam, The Netherlands); Kerri Beckmann (King's College London, London, UK & Guy’s and St Thomas’ NHS Foundation Trust, London, UK), Brian Denton (University of Michigan, Michigan, USA), Andrew Hayen (University of Technology Sydney, Australia), Paul Boutros (Ontario Institute of Cancer Research, Toronto, Ontario, Canada).

Clinical Research Partners’ IT Experts: Wei Guo (Johns Hopkins University, The James Buchanan Brady Urological Institute, Baltimore, USA), Nicole Benfante (Memorial Sloan Kettering Cancer Center, New York, USA), Janet Cowan (University of California San Francisco, San Francisco, USA), Dattatraya Patil (Emory University School of Medicine, Winship Cancer Institute, Atlanta, USA), Lauren Park (MD Anderson Cancer Centre, Houston, Texas, USA), Stephanie Ferrante (University of Michigan and Michigan Urological Surgery Improvement Collaborative, Ann Arbor, Michigan, USA), Alexandre Mamedov (University of Toronto, Sunnybrook Health Sciences Centre, Toronto, Ontario, Canada), Vincent LaPointe (University of British Columbia, BC Cancer Agency, Vancouver, Canada), Trafford Crump (University of Calgary, Southern Alberta Institute of Urology, Calgary, Canada), Vasilis Stavrinides (University College London & University College London Hospital Trust, London, UK), Jenna Kimberly-Duffell (University of Cambridge & Cambridge University Hospitals NHS Foundation Trust, Cambridge, UK), Aida Santaolalla (King's College London, London, UK & Guy’s and St Thomas’ NHS Foundation Trust, London, UK), Daan Nieboer (Erasmus Medical Center, Rotterdam, The Netherlands), Jonathan Olivier (Lille University Hospital Center, Lille, & Centre Hospitalier Universitaire de Bordeaux (CHU), Bordeaux France), Tiziana Rancati (Fondazione IRCCS Istituto Nazionale dei Tumori di Milano, Milan, Italy), Helén Ahlgren (Sahlgrenska University Hospital, Göteborg, Sweden), Juanma Mascarós (Instituto Valenciano de Oncología, Valencia, Spain), Annica Löfgren (Skåne University Hospital, Malmö, Sweden), Kurt Lehmann (Kantonsspital Baden, Baden, Switzerland), Catherine Han Lin (Monash University and Epworth HealthCare, Melbourne, Australia), Thomas Cusick (St Vincents Prostate Cancer Centre, Sydney, Australia), Hiromi Hirama (Kagawa University, Kagawa, Japan), Kwang Suk Lee (Yonsei University College of Medicine, Gangnam Severance Hospital, Seoul, Korea).

Research Advisory Committee: Guido Jenster (Erasmus MC, Rotterdam, the Netherlands), Anssi Auvinen (University of Tampere, Tampere, Finland), Anders Bjartell (Skåne University Hospital, Malmö, Sweden), Masoom Haider (University of Toronto, Toronto, Canada), Kees van Bochove (The Hyve B.V. Utrecht, Utrecht, the Netherlands).

Management team: Michelle Kouspou (Movember Foundation, Melbourne, Australia), Kellie Paich (Movember Foundation, Los Angeles, USA), Chris Bangma (Erasmus Medical Center, Rotterdam, The Netherlands), Monique Roobol (Erasmus Medical Center, Rotterdam, The Netherlands), Jozien Helleman (Erasmus Medical Center, Rotterdam, The Netherlands).
