# Supplementary material for: Risk of progression following a negative biopsy in prostate cancer active surveillance
Source: Prostate Cancer Prostatic Dis. 2022 Aug 25;26(2):403–9. doi: 10.1038/s41391-022-00582-x (PMC10247354; doi:10.1038/s41391-022-00582-x)
Supplement: Supplementary file 2 — Supplementary Material [file 41391_2022_582_MOESM2_ESM.docx]

**Supplementary Figure 1. Participant inclusion schema**

**
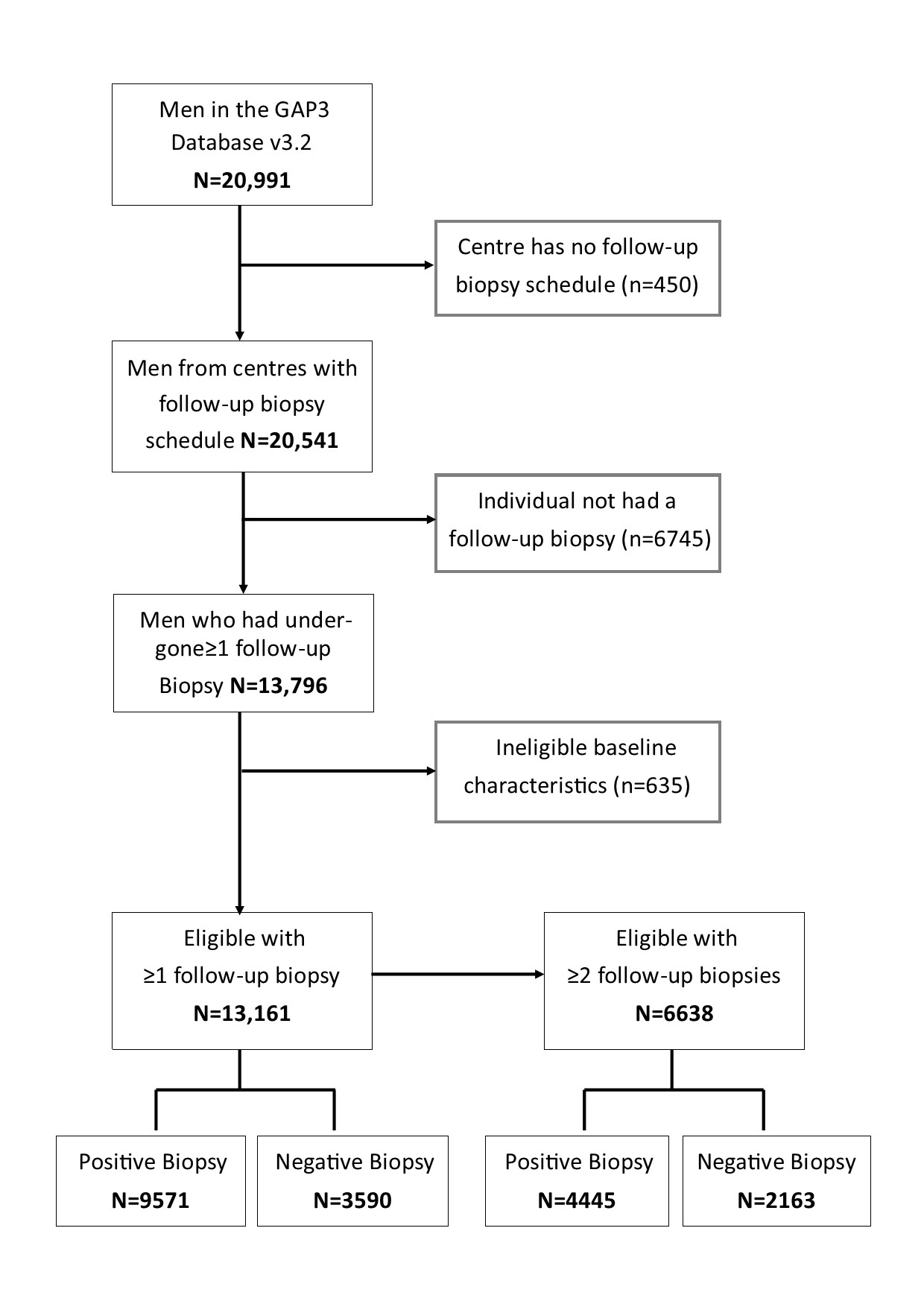
**

**Supplementary Table 1. Risk of transitioning to treatment among men on active surveillance for prostate cancer**

|  | **All eligible men**  **(n=13161)** | | | **Men with ≥1 subsequent follow-up biopsy**  **(n=6138)** | | |
| --- | --- | --- | --- | --- | --- | --- |
| **Factors** | **HR** | **95% CI** | **p-value** | **HR** | **95% CI** | **p-value** |
| Negative biopsy [vs positive biopsy] | 0.45 | 0.42-0.49 | <0.001 | 0.65 | 0.59-0.72 | <0.001 |
| Age [Ref: <55yrs] | 1.00 | - | - | 1.00 | - | - |
| 55-59 | 1.11 | 0.98-1.25 | 0.11 | 1.15 | 0.98-1.37 | 0.10 |
| 60-64 | 1.20 | 1.07-1.35 | 0.002 | 1.19 | 1.01-1.40 | 0.033 |
| 65-69 | 1.25 | 1.11-1.40 | <0.001 | 1.22 | 1.04-1.44 | 0.014 |
| 70-74 | 1.18 | 1.05-1.34 | 0.007 | 1.13 | 0.95-1.35 | 0.17 |
| ≥75 | 1.11 | 0.95-1.29 | 0.19 | 1.02 | 0.74-1.31 | 0.88 |
| Diagnosis period [Ref: <2004] | 1.00 | - | - | 1.00 | - | - |
| 2005-2009 | 1.10 | 0.97-1.24 | 0.13 | 1.16 | 0.98-1.37 | 0.081 |
| 2010-2014 | 1.43 | 1.26-1.61 | <0.001 | 1.38 | 1.16-1.63 | <0.001 |
| 2015-2018 | 1.84 | 1.05-2.12 | <0.001 | 1.67 | 1.36-2.04 | <0.001 |
| Diagnostic PSA [Ref: <5ng/ml] | 1.00 | - | - | 1.00 | - | - |
| 5-<10 | 1.35 | 1.27-1.44 | <0.001 | 1.35 | 1.23-1.48 | <0.001 |
| 10-<15 | 1.49 | 1.29-1.72 | <0.001 | 1.32 | 1.07-1.64 | 0.011 |
| 15-<20 | 1.51 | 1.14-1.99 | 0.004 | 1.40 | 0.95-2.04 | 0.087 |
| Grade dx [3+4 *vs* 3+3] | 1.17 | 0.98-1.30 | 0.026 | 1.43 | 1.16-1.76 | 0.001 |
| Stage dx [cT2 *vs* cT1] | 1.15 | 1.07-1.25 | <0.001 | 1.08 | 0.97-2.07 | 0.15 |
| Cores taken dx [continuous] | 0.988 | 0.975-0.989 | <0.001 | 0.988 | 0.977-0.996 | 0.004 |
| Cores positive dx [continuous] | 1.10 | 1.07-1.12 | <0.001 | 1.08 | 1.06-1.11 | <0.001 |
| Prostate volume [cc] | 0.989 | 0.987-0.990 | <0.001 | 0.99 | 0.986-0.991 | <0.001 |
| Interval to first biopsy [years] | 0.94 | 0.91-0.96 | <0.001 | 1.13 | 1.08-1.19 | <0.001 |
|  |  |  |  |  |  |  |

HR: Hazard ratios derived from mixed -effects survival regression (survival time from first biopsy date), with Weibull distribution and random intercept for treatment centre, adjusted simultaneously for age, PSA, grade group, CT-stage, no. core sampled, no. cores positive, prostate volume, time interval between diagnosis and first follow-up biopsy.

**Supplementary Table 2. Risk of subsequent upgrading and upgrading to Grade Group >2 among men who underwent 2 or more biopsies while on active surveillance for prostate cancer**

|  | **Any upgrading** | | | **Upgraded to grade group>2** | | |
| --- | --- | --- | --- | --- | --- | --- |
| **Factors** | **0R** | **95% CI** | **p-value** | **0R** | **95% CI** | **p-value** |
| Negative biopsy [vs positive biopsy] | 0.52 | 0.45-0.60 | <0.001 | 0.74 | 0.59-0.92 | 0.007 |
| Age [Ref: <55yrs] | 1.00 | - | - | 1.00 | - | - |
| 55-59 | 0.88 | 0.70-1.10 | 0.272 | 0.98 | 0.71-1.36 | 0.919 |
| 60-64 | 0.86 | 0.70-1.07 | 0.179 | 0.95 | 0.68-1.27 | 0.653 |
| 65-69 | 1.07 | 0.86-1.32 | 0.548 | 1.19 | 0.87-1.61 | 0.272 |
| 70-74 | 1.15 | 0.91-1.45 | 0.247 | 1.42 | 1.02-1.97 | 0.038 |
| ≥75 | 1.12 | 0.81-1.55 | 0.481 | 1.55 | 1.01-2.41 | 0.045 |
| Diagnosis period [Ref: <2004] | 1.00 | - | - | 1.00 | - | - |
| 2005-2009 | 0.71 | 0.55-0.92 | 0.009 | 0.73 | 0.52-1.02 | 0.077 |
| 2010-2014 | 0.73 | 0.57-0.95 | 0.020 | 0.78 | 0.56-1.10 | 0.154 |
| 2015-2018 | 0.69 | 0.52-0.92 | 0.011 | 0.75 | 0.51-2.41 | 0.135 |
| Diagnostic PSA [Ref: <5ng/ml] | 1.00 | - | - | 1.00 | - | - |
| 5-<10 | 1.03 | 0.91-1.17 | 0.671 | 1.10 | 0.93-1.22 | 0.290 |
| 10-<15 | 0.89 | 0.67-1.18 | 0.417 | 1.01 | 0.68-1.51 | 0.954 |
| 15-<20 | 0.90 | 0.52-1.56 | 0.713 | 1.35 | 0.69-2.67 | 0.384 |
| Grade dx [3+4 *vs* 3+3] | 0.25 | 0.17-0.25 | <0.001 | 1.15 | 0.80-1.65 | 0.449 |
| Stage dx [cT2 *vs* cT1] | 1.02 | 0.87-1.19 | 0.831 | 1.14 | 0.92-1.40 | 0.232 |
| Cores taken dx [continuous] | 1.00 | 0.99-1.01 | 0.838 | 0.99 | 0.98-1.01 | 0.508 |
| Cores positive dx [continuous] | 1.07 | 1.02-1.12 | 0.003 | 1.01 | 0.96-1.07 | 0.664 |
| Prostate volume [cc] | 1.00 | 0.99-1.00 | 0.044 | 1.00 | 0.99-1.00 | 0.138 |
| Interval to first biopsy [years] | 1.09 | 1.00-1.19 | 0.032 | 1.04 | 0.92-1.16 | 0.540 |
| Interval to second biopsy [years] | 1.00 | 0.96-1.05 | 0.951 | 1.07 | 1.00-1.13 | 0.036 |

OR: Odds ratios derived from mixed effects logistic regression models, with random intercept for treatment centre, among men who had at least two follow-up biopsies (single record per individual, n=6638 men), adjusted simultaneously for age, PSA, grade group, CT-stage, no. core sampled, no. cores positive, prostate volume, time interval between diagnosis and first follow-up biopsy, and time interval between 1^st^ and 2^nd^ biopsy.

**Supplementary Table 3. Odds of subsequent volume progression /volume and grade progression among men who had ≥1 subsequent follow-up biopsy while on active surveillance for prostate cancer**

|  | **Volume progression** | | | **Any upgrading or volume progression** | | |
| --- | --- | --- | --- | --- | --- | --- |
| **Factors** | **0R** | **95% CI** | **p-value** | **0R** | **95% CI** | **p-value** |
| Negative biopsy [vs positive biopsy] | 0.34 | 0.28-0.42 | <0.001 | 0.43 | 0.37-0.49 | <0.001 |
| Age [Ref: <55yrs] | 1.00 | - | - | 1.00 | - | - |
| 55-59 | 1.02 | 0.79-1.33 | 0.84 | 1.01 | 0.81-1.26 | 0.92 |
| 60-64 | 1.00 | 0.78-1.28 | 0.98 | 0.97 | 0.78-1.19 | 0.75 |
| 65-69 | 1.04 | 0.81-1.33 | 0.78 | 1.11 | 0.90-1.39 | 0.33 |
| 70-74 | 1.17 | 0.90-1.54 | 0.24 | 1.18 | 0.94-1.49 | 0.14 |
| ≥75 | 1.35 | 0.94-1.94 | 0.10 | 1.23 | 0.90-1.68 | 0.19 |
| Diagnosis period [Ref: <2004] | 1.00 | - | - | 1.00 | - | - |
| 2005-2009 | 0.94 | 0.69-1.27 | 0.67 | 0.76 | 0.59-0.98 | 0.034 |
| 2010-2014 | 0.99 | 0.73-1.34 | 0.94 | 0.78 | 0.60-1.00 | 0.053 |
| 2015-2018 | 1.08 | 0.77-1.49 | 0.67 | 0.74 | 0.56-0.98 | 0.037 |
| Diagnostic PSA [Ref: <5ng/ml] | 1.00 | - | - | 1.00 | - | - |
| 5-<10 | 1.05 | 0.91-1.22 | 0.49 | 0.99 | 0.87-1.11 | 0.82 |
| 10-<15 | 1.00 | 0.73-1.37 | 0.99 | 0.87 | 0.66-1.14 | 0.31 |
| 15-<20 | 0.48 | 0.27-0.96 | 0.038 | 0.64 | 0.38-1.10 | 0.11 |
| Grade dx [3+4 *vs* 3+3] | 1.10 | 0.82-1.49 | 0.53 | 0.71 | 0.53-0.95 | 0.020 |
| Stage dx [cT2 *vs* cT1] | 1.07 | 0.90-1.28 | 0.43 | 1.00 | 0.86-1.17 | 098 |
| Cores taken dx [continuous] | 1.14 | 1.08-1.19 | <0.001 | 0.99 | 0.98-1.00 | 0.19 |
| Cores positive dx [continuous] | 0.98 | 0.97-1.00 | 0.015 | 1.14 | 1.08-1.20 | <0.001 |
| Prostate volume [cc] | 1.00 | 0.99-1.00 | 0.24 | 1.00 | 0.99-1.00 | 0.08 |
| Interval to first biopsy [years] | 1.00 | 0.92-1.09 | 0.99 | 1.05 | 0.98-1.13 | 0.17 |
| Interval to second biopsy [years] | 1.05 | 1.00-1.11 | 0.047 | 0.97 | 0.93-1.01 | 0.17 |

OR: Odds ratios derived from mixed effects logistic regression models, with random intercept for treatment centre, among men who had at least two follow-up biopsies (single record per individual, n=6638 men), adjusted simultaneously for age, PSA, grade group, CT-stage, no. core sampled, no. cores positive, prostate volume, time interval between diagnosis and first follow-up biopsy, and time interval between 1^st^ and 2^nd^ biopsy.

**Supplementary Table 4. Characteristics of men who experienced subsequent upgrading, among those with negative findings at first follow-up biopsy (and ≥1 subsequent biopsy)**

|  | **No upgrading**  1785 (72%) | | **Any subsequent upgrading**  379 (18%) | | **Upgrading to**  **grade group >2**  149 (7%) | |
| --- | --- | --- | --- | --- | --- | --- |
| **Diagnostic characteristics:** |  |  |  |  |  |  |
| Median age (IQR) | 64 | (59-68) | 65 | (61-69) | 66 | (61-70) |
| Median PSA (IQR) | 5.3 | (4.0-7.0) | 5.1 | (4.1-6.6) | 5.1 | (4.1-6.7) |
| Grade (n, %) |  |  |  |  |  |  |
| grade group 1 | 1768 | (99) | 374 | (99) | 144 | (97) |
| grade group 2 | 16 | (1) | 5 | (1) | 5 | (3) |
| Clinical T stage (n, %) |  |  |  |  |  |  |
| cT1 | 1532 | (86) | 332 | (88) | 125 | (84) |
| cT2 | 252 | (14) | 47 | (12) | 24 | (16) |
| Median no. cores sampled | 12 | (10-12) | 12 | (10-12) | 12 | (10-12) |
| Median no. cores positive | 1 | (1-2) | 1 | (1-2) | 1 | (1-2) |
| Median prostate volume, cc (IQR) | 48 | (37-60 | 46 | (35-57) | 47 | (38-57) |
| Median PSA density, (IQR) | 0.111 | (0.078-0.148) | 0.117 | (0.085-0.153) | 0.116 | (0.087-0.148) |
| PSA density group: |  |  |  |  |  |  |
| <0.10 | 735 | (41) | 138 | (36) | 52 | (35) |
| 0.10-0.149 | 615 | (35) | 139 | (37) | 54 | (36) |
| 0.15-0.199 | 341 | (19) | 82 | (22) | 28 | (19) |
| ≥0.20 | 93 | (5) | 20 | (5) | 15 | (10) |
| **Characteristic at 1^st^ FU biopsy** |  |  |  |  |  |  |
| Median FU PSA, (IQR) | 4.9 | 3.1-6.8 | 5.4 | 3.8-7.4 | 5.4 | 3.9-7.4 |
| Median FU cores sampled, (IQR) | 12 | 10-14 | 12 | 10-14 | 12 | 10-12 |
| FU PSA density, (IQR) | 0.10 | 0.06-0.14 | 0.12 | 0.08-0.17 | 0.12 | 0.08-0.17 |
| Median years to 1^st^ biopsy, (IQR) | 1.0 | 0.6-1.1 | 1.0 | 0.6-1.1 | 1.0 | 0.6-1.2 |
| Median years to upgrade, (IQR) | - | - | 4.1 | 2.8-5.8 | 4.0 | 2.6-6.0 |
|  |  |  |  |  |  |  |
